# Polymerised mite allergoids with glutaraldehyde reduce proteolytic activity and enhance the stability of allergen mixtures: a proof of concept with grass mixtures

**DOI:** 10.3389/falgy.2025.1557650

**Published:** 2025-02-25

**Authors:** José Ignacio Tudela, Irene Soria, Eva Abel-Fernández, José Fernando Cantillo, Enrique Fernández-Caldas, José Luis Subiza, Salvador Iborra

**Affiliations:** ^1^ R&D Department, Inmunotek, Alcalá de Henares, Spain; ^2^ R&D Department, Fundación Inmunotek, Alcalá de Henares, Spain

**Keywords:** allergen immunotherapy (AIT), polymerised allergens, grass pollen allergens, house dust mite, proteolytic activity, multi-allergen vaccines, polysensitized patients

## Abstract

**Background:**

Polysensitized patients require allergen immunotherapy (AIT) targeting multiple allergens. However, combining allergen extracts can lead to instability and reduced efficacy particularly due to the high proteolytic activity of house dust mite (HDM) allergens. While is known that glutaraldehyde cross-linking may reduce enzymatic activity, its ability to stabilize multi-allergen formulations and protect key allergens from degradation remains unexplored.

**Objective:**

To evaluate the impact of glutaraldehyde polymerization on the stability and immunogenicity of HDM and grass pollen allergen formulations, addressing proteolytic activity challenges in multi-allergen vaccines.

**Methods:**

Stability was assessed over 24 months through protein quantification and antigenic activity assays. Proteolytic activity of HDM-containing extracts was measured using Azocoll, and peptide substrate-based enzymatic assays. Grass pollen allergen degradation was evaluated by SDS-PAGE, immunoblotting, and ELISA Immunogenicity was assessed in mice immunized with grass allergoids alone or in combination with glutaraldehyde-polymerised HDM, measuring IgG responses, splenocyte proliferation, and IL-10 production.

**Results:**

Glutaraldehyde polymerization significantly reduced HDM proteolytic activity (*p* < 0.0001), achieving reductions of 97.7%, 77.9%, and 89.9% in total protease activity, cysteine protease activity, and serine protease activity, respectively. This inhibition protected grass pollen allergens when mixed with HDM from degradation, ensuring consistent protein content and antigenic activity over 24 months. Mice immunized with grass allergoids alone or combined with polymerised mite extracts showed similar IgG responses and T-cell activation, indicating no compromise in the immune response to grass allergens, with IL-10 secretion confirming preserved regulatory responses.

**Conclusions:**

Polymerised allergen extracts address the challenges of proteolytic degradation in multi-allergen formulations, offering stable, immunogenic vaccines that maintain efficacy and provide a reliable treatment option for polyallergic patients.

## Introduction

Allergen immunotherapy (AIT) is the only disease-modifying treatment for IgE-mediated allergies, offering long-term relief by administering increasing doses of allergens (1, 2). House dust mites (HDM) and grass pollen are among the most prevalent allergens globally, with HDM being a key trigger for allergic asthma, especially in urban areas (3). In Europe, the European Academy of Allergy and Clinical Immunology (EAACI) has recognized HDM as a critical allergen, with guidelines emphasizing its role in allergic asthma management (4). Similarly, grass pollen allergies affect 20%–30% of adults and 40% of children, especially in temperate regions. Prevalence has risen in Mediterranean countries, where *Poaceae* family pollen contributes significantly to rhinitis and asthma (5–7).

Polysensitization, defined as sensitization to multiple allergen sources, has a prevalence ranging from 27.5%–97.4%, depending on the population studied (8–14). Though once considered less effective for polysensitized patients, recent evidence shows AIT benefits both mono- and polysensitized individuals (15, 16). Subcutaneous immunotherapy (SCIT) typically requires long-term administration, with injections every 4–8 weeks for a minimum of three years. These prolonged treatment protocols often result in poor adherence, which can be particularly challenging for polyallergic patients who are polysensitized to clinically relevant allergens. AIT with allergen mixtures offers a solution by addressing multiple allergies simultaneously, potentially improving patient adherence and quality of life.

Developing effective AIT formulations for polyallergic patients is challenging, particularly when combining proteolytic and non-proteolytic allergens. Protease-containing allergens, such as those from HDMs, can degrade other allergens in mixed formulations, reducing vaccine stability and immunogenicity (17–21). For example, cysteine protease (Der p 1) and serine proteases (Der p 3, Der p 6, and Der p 9) from *Dermatophagoides pteronyssinus (D. pteronyssinus)* contribute to disrupting epithelial barriers, promoting Th2 immune responses (20), and exacerbating allergic reactions (22–25). These proteolytic activities can degrade other proteins, including pollen allergens (25), potentially impairing vaccine stability and immune responses.

Current regulatory guidelines pose challenges for multi-allergen formulations. While the FDA permits allergen mixing (https://college.acaai.org/wp-content/uploads/2023/07/ACAAIMixingInstructionGuide2023v5.pdf), the EMA (advises against combining proteolytic allergens like HDMs with grass pollen due to potential degradation (https://www.ema.europa.eu/en/allergen-products-production-quality-issues-scientific-guideline). This lack of harmonization poses challenges and highlights the need for scientifically validated strategies to enhance vaccine stability, a gap this study aims to address by evaluating glutaraldehyde polymerization as a protective approach.

Previous studies shown that chemically modified allergens treated with glutaraldehyde results in highly polymerised structures with reduced allergenicity, making IgE epitopes less accessible. Moreover, glutaraldehyde treatment may inhibit enzyme activity by modifying the catalytic sites (26–28). However, its effectiveness in stabilizing multi-allergen formulations and protecting allergens from enzymatic degradation has not been fully explored, a gap this study aims to address.

This study investigates the proteolytic activity of native and glutaraldehyde-polymerised *D. pteronyssinus* extracts and their effects on grass pollen allergens [*Phleum pratense* (*P. pratense*)]. We also evaluate the stability of a polymerised mite-grass allergen mixture and assess its immunogenicity. These findings could offer valuable insights into AIT formulations with polymerised allergen mixtures used in polyallergic patients.

## Methods

### Native and polymerised allergen extracts

Mite freeze-dried native extracts (MNE) from *D. pteronyssinus* and *Dermatophagoides farinae (D. farinae)* were prepared (Dpt NE Dfa NE respectively)*.* Grass native extracts (GNE) were derived from pollen grains of *Holcus lanatus (H. lanatus), Dactylis glomerata (D. glomerata), Lolium perenne (L. perenne), P. pratense, Poa pratensis (P. pratensis), and Festuca elatior (F. elatior)*. The protein content was quantified using the Bradford assay (Bio-Rad Laboratories, Madrid, Spain). Polymerised grass (GPE) and mite (MPE) extracts were obtained using glutaraldehyde (25%, Sigma-Aldrich) following protocols described previously for *P. pratense* grass pollen (Manzano et al., 2016) and *D. farinae* mites (29).

### Vaccines

Vaccines in this study were produced by Inmunotek S.L. under Good Manufacturing Practice (GMP) standards, and included GPE (*P. pratense, D. glomerata, F. elatior, H. lanatus, L. perenne, P. pratensis*, 10,000 TU/ml or 30,000 TU/ml) alone or mixed with MPE (*D. pteronyssinus* PE combined or not with *D. farinae* PE, 10,000 TU/ml or 30,000 TU/ml each one). All vaccines were produced under sterile conditions, adsorbed to aluminum hydroxide [Al(OH)_3_], and stored at 4°C from 0 day in sealed vials.

### Stability studies

Vaccine vials containing GPE alone (30,000 TU/ml) or combined with MPE (*D. pteronyssinus* and *D. farinae*, 30,000 TU/ml) were stored at 4°C and analyzed at baseline and intervals (3–24 months). Total protein content was measured using the nitrogen Total Organic Carbon (TOC) method with a TOC-TNb MULTI NC 2100s CLD analyzer (Analytik Jena AG), employing the Dumas method at 800°C. Nitrogen dioxide (NO_2_) generated during combustion was quantified via chemiluminescence against a calibration curve.

### ELISA competition assays

Biological potencies (IC_50_) of vaccines and allergens were evaluated by ELISA competition assays. Plates were coated with 1 *µ*g of *P. pratense* NE. For vaccines, assays were performed using grass-specific rabbit IgG. Competing samples were incubated with polyclonal IgG antibodies generated by immunizing rabbits subcutaneously (s.c.) with *P. pratense* allergoids emulsified in Freund's complete adjuvant. After incubation, goat anti-rabbit IgG was added. For analysing the proteolytic effects of *D. pteronyssinus* NE and PE, patient serum-based assays were conducted. Competing samples were incubated with a pool of sera from grass-sensitized patients, followed by incubation with peroxidase-conjugated anti-human IgE antibodies. Reactions were developed using o-phenylenediamine dihydrochloride (OPD; Sigma-Aldrich) as the substrate, stopped with 3.75% HCl, and absorbance was measured at 492 nm. Percent inhibition was calculated as: 100—[(OD with competitor/OD without competitor) × 100]. IC_50_ values were derived using semi-log plots. All samples were tested in duplicate, with non-inhibition and blank controls included.

### Proteolytic activity analysis

Proteolytic activity of *D. pteronyssinus* extracts was measured using trypsin, serine protease, and cysteine protease-specific substrates. Hydrolysis products were quantified spectrophotometrically and expressed as enzyme equivalents based on standard curves. Total protease activity was assessed with Azocoll (Sigma), a dye-impregnated collagen substrate. Samples and trypsin standards were diluted in 10 mM SPB buffer and incubated with Azocoll (6 mg/ml) for 1 h at 37°C. After centrifugation, supernatants were collected, and dye release was measured at 540 nm. Results were expressed as enzyme equivalents per milligram of protein based on the trypsin standard curve. Cysteine protease activity was evaluated using Pyr-Phe-Leu-p-nitroanilide (L-1460) as the substrate and papain (Sigma) as the standard. Samples and standards were diluted in 0.2 M sodium acetate/acetic acid buffer (pH 4.0). L-Cys/EDTA (40 mM) and the substrate were added, and reactions were incubated for 30 min at 37°C. The reaction was stopped with 50% acetic acid, and absorbance was measured at 405 nm. Data were expressed as enzyme equivalents per milligram of protein, based on the papain standard curve. Serine protease activity was determined using D-Ile-Pro-Arg-p-nitroanilide dihydrochloride (S-2288) as the substrate and trypsin (Sigma) as the standard. Reactions were conducted in 0.1 M Tris-HCl buffer (pH 8.4) at 37°C for 30 min and stopped with 50% acetic acid. Absorbance was measured at 405 nm, and enzyme activity was expressed as equivalents per milligram of protein.

Native *D. pteronyssinus* extracts heated at 70°C for 30 min (Dpt HE) served as a positive control for enzymatic inactivation.

### Effect of mite proteolytic activity on grass pollen native extract

**Figure 1:**
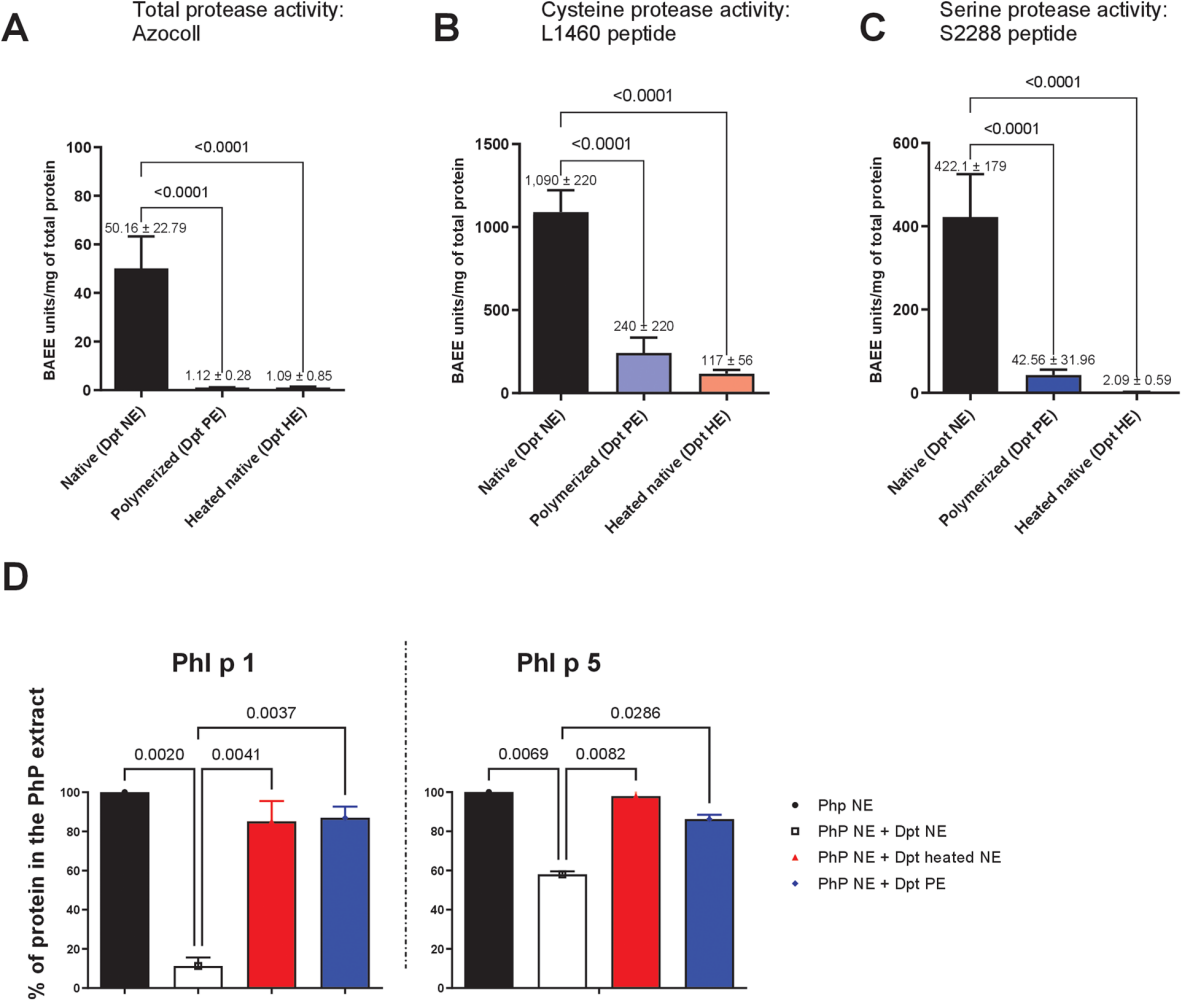
Assessment of proteolytic activity in native and glutaraldehyde-polymerised *D. pteronyssinus* extracts. Total and specific protease activities were measured in native and glutaraldehyde-polymerised *D. pteronyssinus* extracts, with a heated native extract serving as a control. **(A)** Total protease activity was evaluated using the Azocoll-based assay. **(B)** Cysteine protease activity was assessed using the L-1460 peptide substrate. **(C)** Serine protease activity was measured using a specific serine protease peptide substrate (S2888). Error bars represent SEM from the mean. **(D)** ELISA: Quantification of major *P. pratense* allergens in mixtures with *D. pteronyssinus* extracts after 30 min of incubation. Samples were mixed at a final concentration of 500 *µ*g/ml. The graph shows the mean percentage of Phl p 1 and Phl p 5 content in native *P. pratense* extract (Php NE) upon mixing with heated or non-heated native mite extract (Dpt NE) or polymerised extract (Dpt PE) from two independent experiments. Error bars represent SEM from the mean.

**Figure 2:**
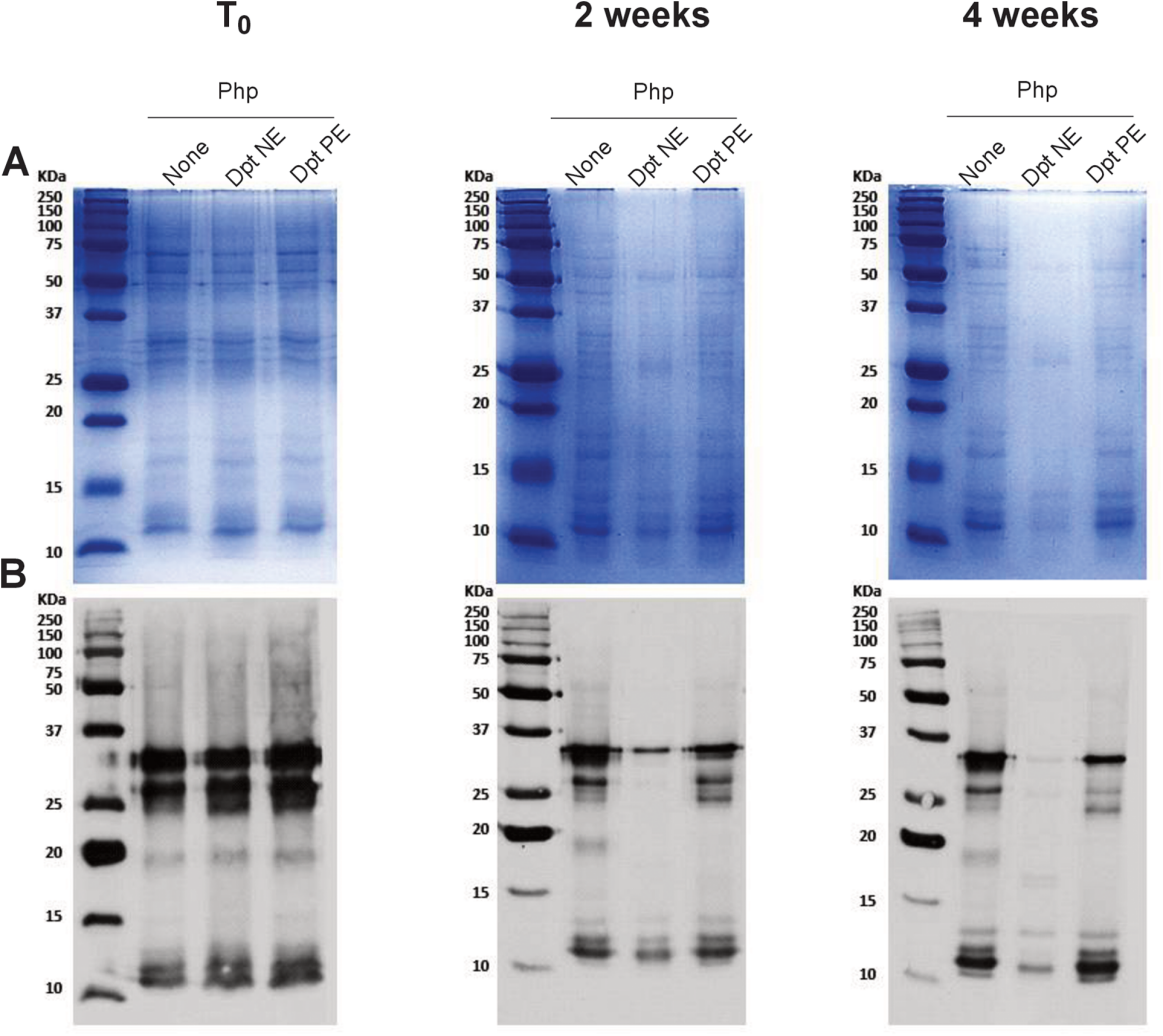
Polymerization of mite allergens reduces their degradative effect on grass pollen allergens. **(A)** Protein profile: SDS-PAGE analysis of *P. pratense* (250 *µ*g/ml) following incubation with *D. pteronyssinus* native extract (NE, 25 *µ*g/ml; lane 2) or polymerised extract (PE, 25 *µ*g/ml; lane 3) over various periods. Lane 1 shows the *P. pratense* protein profile without incubation as a control. **(B)** Allergenic profile: IgE immunoblots showing the allergenic profile of *P. pratense* under the same conditions as in panel **(A)**, comparing the impact of NE and PE on allergen stability.

Native and polymerised *D. pteronyssinus* extracts (Dpt NE, Dpt PE) were mixed with *P. pratense* native extracts (Php NE) in aqueous solution and stored at 4°C. Mixtures were analysed at various time points to evaluate degradation. Two experimental conditions were tested: (1) mixtures containing 500 *µ*g/ml of Php NE with 500 *µ*g/ml of Dpt NE or Dpt PE (Figure 1D, Supplementary Figures S2, S3); (2) mixtures containing 250 *µ*g/ml of Php NE with 25 *µ*g/ml of Dpt NE or Dpt PE (Figure 2).

The degradative effect of *D. pteronyssinus* extracts on *P. pratense* was assessed using SDS-PAGE, immunoblotting, and ELISA competition assays. Major grass allergens, Phl p 1 and Phl p 5, were quantified using commercial kits (Indoor Biotechnologies Ltd). Incubation periods included 2 and 4 weeks for SDS-PAGE and immunoblotting, and 30 min, 8 days, and 20 days for ELISA competition assays.

### SDS-PAGE and immunoblotting

Php NE mixed or not with Dpt NE or Dpt PE were analysed by SDS-PAGE under reducing conditions and immunoblotting using a pool of human serum IgE specific to *P. pratense* as described previously (29).

### Immunization protocol

Female Balb/c mice (7–8 weeks old) (*n* = 7 per group) were immunized subcutaneously three times at 15-day intervals. Seven days after the final immunization, blood and spleens were collected as described below. Animal studies were approved by the local ethics committee (PROEX 169.1/21). All procedures adhered to European Community guidelines (86/609/EEC). Immunizations were conducted at two and eight months after allergen preparation. The groups were: (a) Negative control: Al(OH)₃ alone; (b) Grass PE: PGE (10,000 TU/ml) (c) Grass PE + *Dpt* PE: PGE (10,000 TU/ml) + *D. pteronyssinus* PE (10,000 TU/ml): (d) Grass PE + *Dpt* PE *+* *Dfa* PE: PGE (10,000 TU/ml) + *D. pteronyssinus* PE (10,000 TU/ml) + *D. farinae* PE (10,000 TU/ml). All vaccines were absorbed to Al(OH)_3_. Mice received 200 *µ*l of each preparation.

### Serum IgG levels

Blood was collected via cardiac puncture, centrifuged, and stored at −80°C. Serum IgG was measured by ELISA using native grass extract as the antigen.

### Splenocyte culture, proliferation and IL-10 measurement

Splenocytes were isolated (1 × 10⁶), from mice immunized with allergens prepared 8 months earlier, labelled with 3 *µ*M CFSE (Invitrogen) and cultured at 1 × 10⁶ cells/ml in RPMI-1640 with 10% FCS, gentamicin, and L-glutamine. Stimulation was performed with NGE at 50 *µ*g/ml or controls (complete medium alone or phytohemagglutinin). Proliferation was assessed on days 5–7 using a FC500 flow cytometer (Beckman Coulter). IL-10 levels in supernatants were quantified 48 h post-stimulation using FlowCytomix (eBioscience), with data acquisition on a FC500 flow cytometer and analysis via FlowCytomix-Pro software.

All results were reported as the average of duplicate measurements.

### Statistics

Sample sizes (*n* = 7 per group) were determined based on power calculations to detect biologically relevant differences of approximately 0.75 standard deviations, with 80% power at a significance level of *α* = 0.05. Calculations assumed an expected variability (*σ*) of 0.5, based on prior experimental data. Data are expressed as mean ± SEM. Assumptions of normality (Shapiro–Wilk test) and homogeneity of variances (Levene's test) were verified before applying parametric tests. Statistical analysis was performed using one-way ANOVA, followed by Tukey's *post-hoc* test for multiple comparisons (95% confidence interval), or Student's *t*-test for paired or unpaired comparisons. Significance levels were set at *p* < 0.05.

## Results

### Long-term stability of polymerised grass-mite vaccine formulations

Routine stability assessments confirmed that a multi-allergen vaccine comprising polymerised grass pollen allergens (*P. pratense*, *H. lanatus, D. glomerata, L. perenne*, *P. pratensis*, and *F. elatior*) combined with polymerised mite extracts (*D. pteronyssinus and D. farinae*), met regulatory standards by maintaining consistent protein content and immunogenic activity over 24 months (see Supplementary Figure S1). Stability was observed regardless of whether grass pollen polymers were mixed with polymerised mite extracts or evaluated alone (see Supplementary Figure S1A,B). The total protein content of the vaccine mixture showed minimal variation over time, remaining within acceptable limits for regulatory compliance (see Supplementary Figure S1A,B).

With validated assay limits, an IgG potency test evaluated antigen stability over time. Timothy grass (*P. pratense*) IC_50_ values ranged from 0.621–2.483 *µ*g/ml, starting at 1.68 *µ*g/ml at T0, decreasing slightly to 1.291 *µ*g/ml at T24m (average: 1.257 ± 50%–200%; see Supplementary Figure S1C). The stability of the IC_50_ values for the grass-mite mixture closely matched those of the grass polymers evaluated alone (see Supplementary Figure S1D).

### Reduction of proteolytic activity in *D. pteronyssinus* extract following polymerization with glutaraldehyde

To investigate the mechanisms underpinning vaccine stability, we assessed the proteolytic activity of mite allergens and their impact on grass pollen degradation. Glutaraldehyde, a protein cross-linking agent, may inhibit proteolytic enzymes by reacting with their catalytic sites and/or by steric hindrance due to polymerization. Using the Azocoll assay, we evaluated the protease activity of native *D. pteronyssinus* extracts (NE)—containing cysteine protease Der p 1 and serine proteases Der p 3, Der p 6, and Der p 9—and glutaraldehyde-polymerised extracts (PE). PEs exhibited significantly reduced total proteolytic activity compared to untreated NE, with reductions like heat treatment (1.12 ± 0.28 vs. 50.16 ± 22.79 BAEE units/mg total protein; *p* < 0.0001) (Figure 1A). Cysteine protease activity, measured with the L-1460 peptide substrate, showed marked inhibition after polymerization (1,090 ± 220 vs. 240 ± 220 units/mg total protein; *p* < 0.0001) (Figure 1B). Likewise, serine protease activity assessed using the S228 peptide assay was significantly reduced in polymerised extracts (422.1 ± 179 vs. 42.56 ± 31.96 BAEE units/mg total protein; *p* < 0.0001) (Figure 1C). To further confirm the differential proteolytic activity of NE and PE, we incubated *P. pratense* allergens with either HDM NE or PE for 30 min and quantified the allergen levels. When incubated with HDM NE, substantial degradation of *P. pratense* allergens was observed, with Phl p 1 levels reduced tenfold and Phl p 5 levels reduced by 20%. In contrast, both allergens remained intact when incubated with polymerised mite extracts (PE), indicating protection from proteolytic degradation (Figure 1D). Der p 1 and Der p 2 levels remained unaffected, indicating selective degradation of grass pollen allergens without impacting mite allergens (see Supplementary Figure S2).

These findings confirm that glutaraldehyde polymerization effectively inhibits the proteolytic activity of *D. pteronyssinus* extracts, reducing their capacity to degrade grass pollen allergens in mixed formulations.

### Polymerization of HDM allergens reduces grass pollen allergen degradation compared to native extract

Results from SDS-PAGE (Figure 2A) and IgE immunoblot analyses (Figure 2B) revealed that *D. pteronyssinus* native extract degrades *P. pratense* proteins and IgE epitopes, while this degradation is slower in mixtures containing polymerised mite allergoids (Figure 2A,B). Specifically, *P. pratense* IgE-reactive protein bands began to disappear after just one week of incubation with the native mite extract, indicating swift proteolytic activity. In contrast, the *P. pratense* proteins remained more stable in the presence of mite PE allergoids, with bands that may correspond to Phl p 5 (32 kDa), persisting beyond the initial 3 months of incubation.

**Figure 3:**
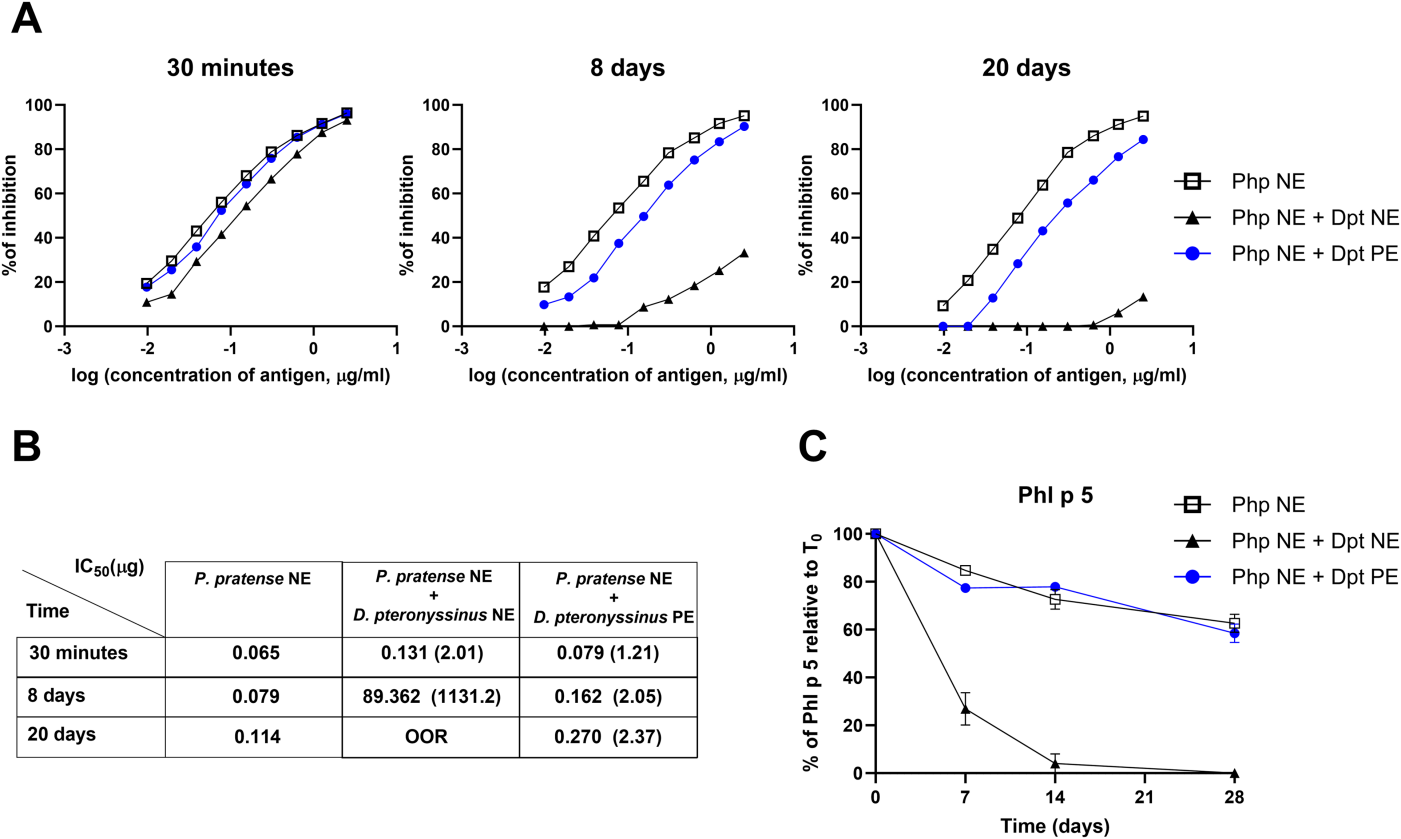
Quantification of mite extract degradation on the IgE biological activity of *P. pratense* native extract over time. Samples (Php NE and Dpt NE or Dpt PE) were mixed at a final concentration of 500 *µ*g/ml. **(A)** Competition ELISA showing the percentage inhibition of specific IgE binding using rabbit sera immunized with *P. pratense* native extract (Php NE). Inhibitors include: Php NE alone (black squares), Php NE combined with *D. pteronyssinus* polymerised extract (PE) (blue circles), and Php NE combined with D. *pteronyssinus* native extract (NE) (black triangles). Measurements were taken at 30 min, 8 days, and 20 days post-incubation. **(B)** Table of IC_50_ values (*µ*g/ml) for each condition at corresponding time points. Lower IC_50_ values indicate greater inhibitory potency, with fold increases in IC_50_ compared to Php NE alone shown in parentheses; >OOR (above out-of-range). **(C)** Phl p 5 levels were quantified and expressed as a percentage relative to the initial amount at time 0 (T_0_) in Php NE alone, (open squares), mixed with Dpt PE (blue circles), or with Dpt NE (black triangles). Data are shown as mean ± standard error (SE) from replicate experiments.

To further confirm these findings, we performed competition ELISA assays (Figure 3A), that showed a significant decrease in the allergenicity of *P. pratense* when mixed with the native mite extract. The inhibitory concentration (IC_50_) values increased sharply, from 0.131 *µ*g at 30 min to 89.32 *µ*g by day 8, exceeding out-of-range levels after 20 days of incubation (Table in Figure 3B). In contrast, when *P. pratense* was incubated with polymerised mite allergoids, the reduction in allergenicity was less pronounced, with IC_50_ values increasing two times after 8 days of incubation, and mildly increasing to 2.37 by day 20. These results indicate that the polymerised *D. pteronyssinus* extract has a milder degrading effect on grass pollen allergens than NE.

Consistent with ELISA results at short incubation periods (Figure 1C), which showed a rapid tenfold reduction in Phl p 1 and only a mild decrease in Phl p 5, quantifying these allergens over longer periods reveals distinct stability patterns. Phl p 5 remains relatively stable over 28 days in Php NE or when incubated in a mixture with Dpt PE (Figure 3C). However, in the presence of Dpt NE, Phl p 5 undergoes rapid degradation, with levels dropping to undetectable amounts by day 14. In contrast, Phl p 1 experiences substantial degradation under all tested conditions, but with no observable differences between its stability in Php NE alone or in mixtures with Dpt PE (see Supplementary Figure S2A). Der p 1 and Der p 2 levels remained unaffected in these mixtures, indicating selective degradation of grass pollen allergens without impacting mite allergens (see Supplementary Figure S2B).

These findings highlight the complementary role of ELISA and immunoblotting in assessing allergen stability. While ELISA provides a quantitative evaluation of antigen levels, immunoblots allow for the visualization of allergen degradation, offering a more comprehensive stability assessment.

### The immunogenicity of grass allergoids remains consistent whether mixed with polymerised mite extracts or used alone

Mixing polymerised mite and grass allergens reduces the degradative effects of mite proteases. Polymerization also enhances grass allergen extracts' safety, stability, and immunogenicity (30, 31). Since using polymerised grass reflects real-world practices, we assessed the immune response elicited by glutaraldehyde polymerised grass allergoids (*H. lanatus, D. glomerata, L. perenne, P. pratense, P. pratensis, and F. elatior*) when administered alone or combined with polymerised mite extracts (*D. pteronyssinus* and *D. farinae*). Mice were immunized with polymerised grass allergoids adsorbed to aluminium hydroxide, either alone or in a grass-mite mixture, with mixtures prepared at two time points (2 and 8 months) before immunization, as described in Materials and Methods.

**Figure 4:**
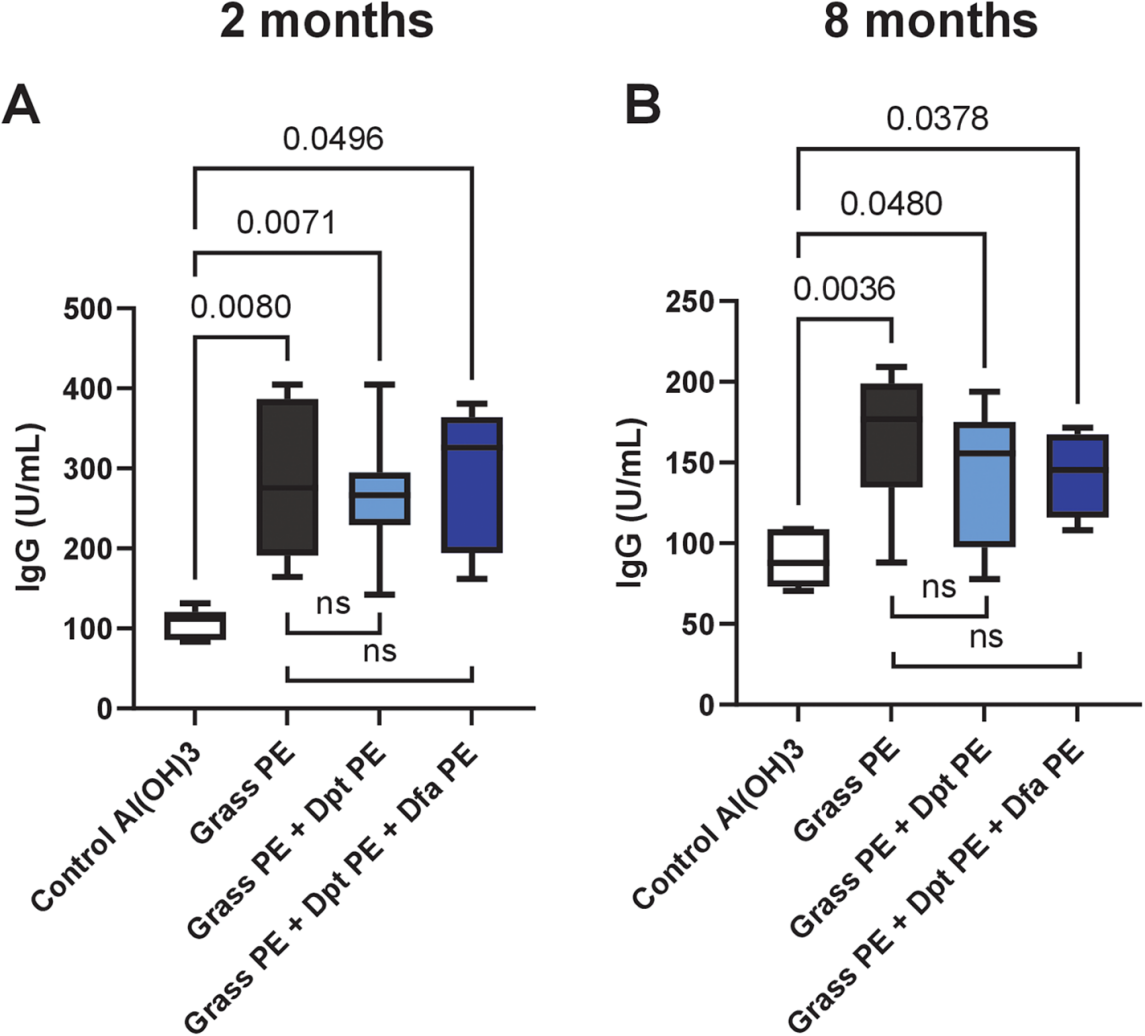
Specific IgG levels against native grass extract in mice immunized with grass allergoids, with or without mite polymers. Mice were immunized with: (1) polymerised extract of six grass species (Grass PE), (2) Grass PE + polymerised *D. pteronyssinus* extract (Dpt PE), or (3) Grass PE + polymerised *D. pteronyssinus* and *D. farinae extracts* (Dpt PE + Dfa PE). IgG levels were assessed at two months **(A)** and eight months **(B)** after preparation of the extracts or extract mixtures. Data are shown as box-and-whisker plots.

As shown in Figure 4, mice immunized with grass polymerised allergoids produced significant IgG responses against grass allergens, unlike non-immunized controls Importantly, there were no significant differences in IgG levels between mice immunized with grass alone and those receiving the grass-mite mixture, regardless of the preparation time (2 months, Figure 4A; 8 months, Figure 4B), indicating that polymerised mite extracts do not affect humoral responses to grass allergens.

**Figure 5:**
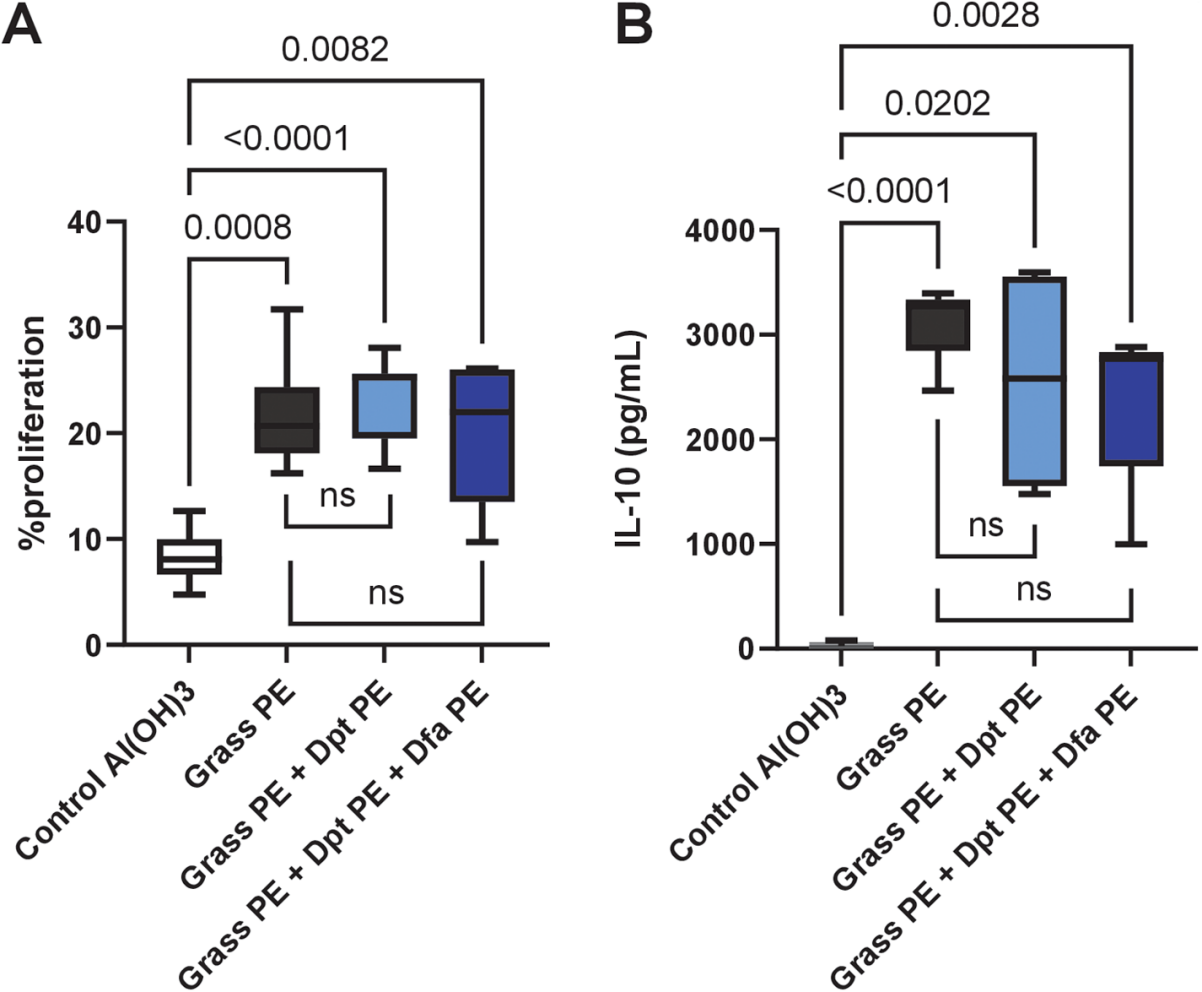
Grass pollen-specific splenocyte proliferation and cytokine release in mice immunized with grass allergoids prepared 8 months earlier, with or without polymerised mite extracts. Mice were immunized with: (1) polymerised extract of six grass species (Grass PE), (2) Grass PE + polymerised *D. pteronyssinus* extract (Dpt PE), or (3) Grass PE + polymerised *D. pteronyssinus* and *D. farinae* extracts (Dpt + Dfa PE). **(A)** Splenocyte proliferation was measured after restimulation with 50 *µ*g/ml of grass pollen NE. **(B)** IL-10 release in splenocyte culture supernatants was also assessed following restimulation with 50 *µ*g/ml of grass pollen NE. Data are presented as box-and-whisker plots.

Similarly, spleen cell proliferation (Figure 5A) in response to grass allergens was comparable across all immunized groups, with allergoids prepared 8 months earlier, with no significant differences between grass-alone and grass-mite groups. IL-10 secretion (Figure 5B), an indicator of regulatory immune responses, was also consistent among all immunized groups, highlighting that the vaccine's ability to induce immune tolerance remains intact when polymerized mite extracts are included.

Overall, these findings demonstrate that co-administration of polymerised mite and grass allergoids in a single preparation, preserves both humoral and cellular immune responses, supporting their combined use in allergen immunotherapy.

## Discussion

Proteolytic allergens, particularly those from house dust mites (HDM), are crucial in initiating allergic responses by enhancing allergen penetration, activating inflammatory pathways, and overcoming airway tolerance (23, 32–34). Group 1 HDM allergens, such as cysteine peptidases, not only sensitize individuals to themselves but also to unrelated allergens, making them important therapeutic targets (23, 35). Inhibitors (35), and engineered recombinant allergens designed to retain immunogenicity while reducing proteolytic activity have been developed (36). Structural modifications to enhance allergen stability can promote a more balanced immune response, favouring regulatory T cell induction and a shift from a Th2 to a Th1 response (37, 38), leading to reduced IgE levels and an increase in IgG4, which is associated with clinical tolerance (39). Inactivating proteolytic allergens could enhance the safety and effectiveness of therapies by reducing allergic reactions and side effects.

Our routine stability assessments demonstrate that the polymerised grass-mite mixture's total protein content and potency as measured by specific IgG binding remained stable over a 24-month. IC_50_ values from IgG competition assays indicated consistent inhibition levels for mite and grass components, suggesting that the vaccine mixture preserved its antigenic properties over time. Here, we confirm the presence of proteolytic activity in native (unmodified) HDM extracts (40) and demonstrate that polymerization of *D. pteronyssinus* allergens significantly reduces this activity, as assessed by the Azocoll assay and specific protease assays. Both cysteine (Der p 1) and serine proteases (Der p 3, Der p 6, and Der p 9) exhibited markedly reduced activity in polymerised extracts compared to native extracts. This aligns with previous studies showing that glutaraldehyde, a cross-linking agent, inhibits enzymes by modifying their catalytic sites (26–28). Polymerised mite extracts demonstrated a significantly reduced capacity to degrade proteins in allergen mixtures, preserving the structural integrity of grass pollen allergens over time. Notably, allergen polymerization with glutaraldehyde achieved a ∼5-fold reduction in cysteine protease activity and a ∼10-fold reduction in serine protease activity. When grass pollen allergens were mixed with native *D. pteronyssinus* extracts, rapid degradation of major allergens occurred within the first two weeks, as demonstrated by SDS-PAGE, immunoblot analysis, and confirmed by ELISA at 20 days. In contrast, when grass pollen allergens were combined with polymerised mite extracts, the allergens remained stable, with proteins intact for over one month of incubation. The substantial reduction in proteolytic activity (97.77% decrease in total protease activity) is clinically significant, as protease-rich allergens—particularly those from HDMs—can degrade co-administered allergens in multi-allergen formulations, potentially compromising the efficacy of AIT (18). By preventing allergen degradation, our findings suggest that polymerization may enhance vaccine stability and potency, ultimately improving clinical outcomes for polysensitized patients undergoing AIT.

A recent study (41), stressed that commercial HDM allergoid vaccines showed high levels of cysteine protease, comparable to that found in native preparations. Here we show that this is not the case when polymerised *Dpt* is compared with the corresponding native counterpart, even when a residual cysteine protease is detected in the polymerised extract. However, variability in polymerization efficacy must be considered. Differences in polymerization efficiency, extract preparation, or cross-linking conditions could introduce batch-to-batch variations in protease inhibition and allergen stability. To address this, we have implemented standardized quality control measures to monitor polymerization consistency, as shown in Supplementary Figure S1. These measures ensure that protease inhibition and allergen stability are maintained across batches, minimizing variability in large-scale vaccine production. While our results demonstrate strong stability under controlled conditions, further studies, including human clinical trials, will be necessary to confirm the efficacy and safety of polymerized allergen formulations in real-world immunotherapy settings. Establishing long-term immune responses and clinical outcomes will be essential for validating their application in AIT. We assessed the immunogenicity and long-term stability of a vaccine mixture containing polymerised grass and mite allergens. Notably, the inclusion of polymerised mite extracts even combined at a twofold concentration (*D. pteronyssinus* and *D. farinae*), did not alter the immunogenicity of grass-polymerised allergoids. Immunization with grass allergoids alone or in combination with polymerised mite extracts without a dilution effect, i.e., maintaining equivalent grass concentration, exhibited comparable IgG responses to native grass allergens, indicating that polymerised mite extracts do not impair the immunogenicity of grass allergens. This was further supported by spleen cell proliferation assays, which suggest similar grass-specific T cell activation across all groups. Additionally, IL-10 production specific for native grass allergens, a marker of regulatory immune responses, remained unaffected by the presence of mite extracts. This preservation of immunogenicity despite protease inhibition may be attributed to the structural integrity of polymerized allergens, which retain essential epitopes for effective immune recognition. Furthermore, reduced enzymatic degradation likely contributes to more consistent antigen presentation.

Regulatory agencies take different stances on multi-allergen formulations, with the FDA permitting mixed extracts under controlled conditions, while the EMA discourages combining proteolytic and non-proteolytic allergens due to stability concerns. Our findings support polymerization as a strategy to enhance stability and immunogenicity, suggesting its potential for revising regulatory restrictions and improving AIT formulations.

Given these regulatory challenges, several stabilization strategies have been developed for multi-allergen AIT, including glycerin-based formulations and protease inhibitors. Glycerin stabilizers effectively inhibit proteases and maintain allergen integrity but require specific storage conditions and may not be universally effective across all allergen classes. Protease inhibitors, while capable of targeting specific enzymes, may not protect against the full range of proteolytic activity present in allergen extracts. In contrast, glutaraldehyde polymerization provides a broader and more robust approach by structurally modifying proteolytic allergens. Additionally, this modification enables the administration of higher doses in a shorter timeframe, potentially improving treatment adherence and efficacy.

## Conclusion

Our study demonstrates that grass allergen mixtures combined with glutaraldehyde-polymerised HDM allergen extracts exhibit excellent stability and preserved immunogenicity, accompanied by a significant reduction in proteolytic activity.

These findings support the use of polymerised extracts to develop stable and effective AIT vaccines for polyallergic patients, minimizing allergen degradation and adverse reactions (42). Enhanced safety allows the combination of polymerised allergens without dilution effect, ensuring the same concentration of single vaccines but with fewer injections and better performance for patient compliance.

## Data Availability

The original contributions presented in the study are included in the article/Supplementary Material, further inquiries can be directed to the corresponding author.
