# Supplementary material for: Polymerised mite allergoids with glutaraldehyde reduce proteolytic activity and enhance the stability of allergen mixtures: a proof of concept with grass mixtures
Source: Front Allergy. 2025 Feb 25;6:1557650. doi: 10.3389/falgy.2025.1557650 (PMC11893842; doi:10.3389/falgy.2025.1557650)
Supplement: Supplementary file 1 [file Datasheet1.docx]

**Polymerised mite allergoids with glutaraldehyde reduce proteolytic activity and enhance the stability of allergen mixtures: a proof of concept with grass mixtures**

Brief running title: Protease activity in mixed allergen extracts

**(Supplemental figures)**

**
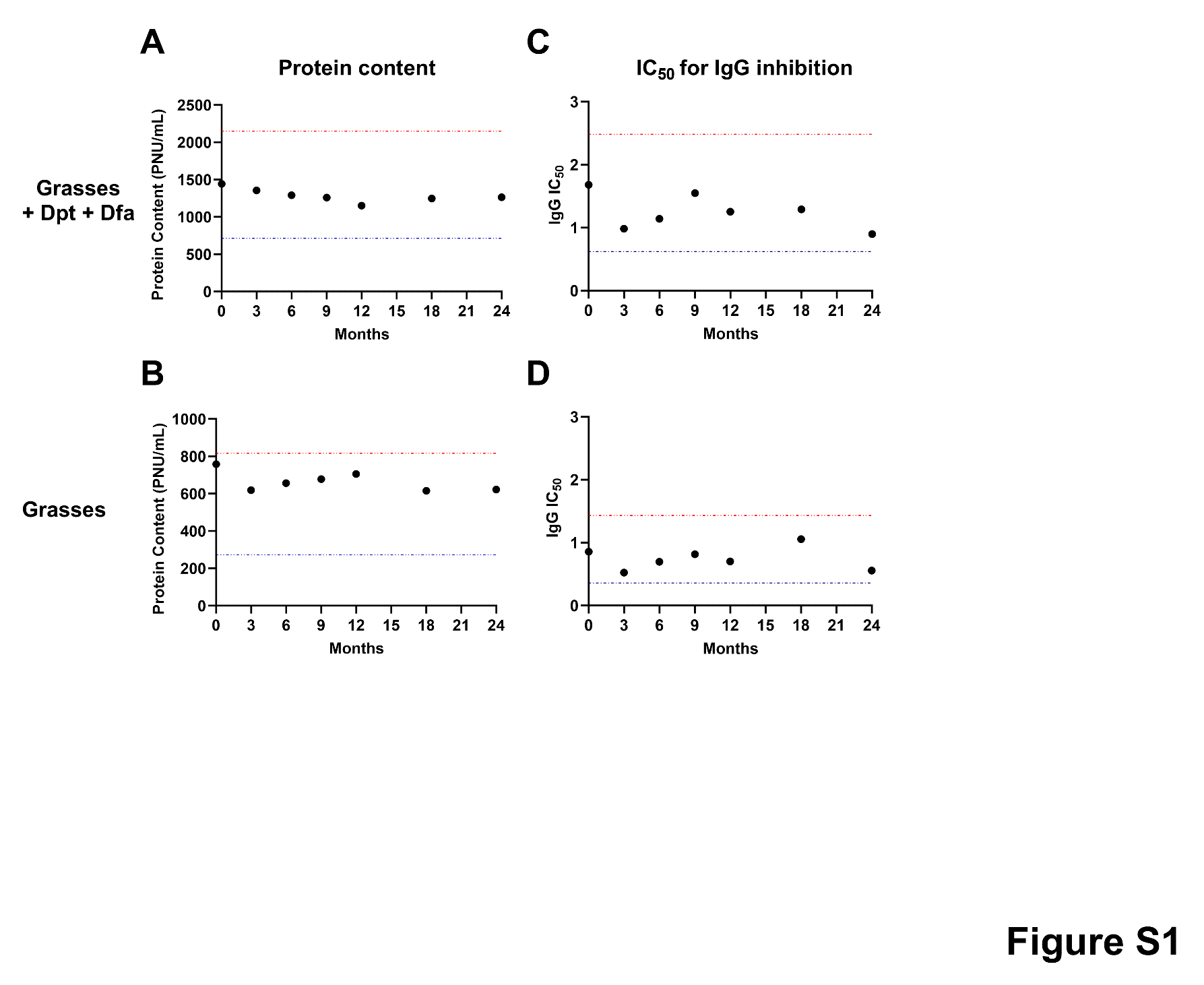
**

**Figure S1. Stability profile of vaccine components over 24 months.** The stability of the vaccine formulation was evaluated at multiple time points (0, 3, 6, 9, 12, 18, and 24 months) for several parameters. Panels (A) and (B) show the protein content, expressed in Protein Nitrogen Units (PNU/mL), of the combined formulation (grasses + Dpt + Dfa) and grasses alone, respectively. Panels (C) and (D) depict the IC₅₀ values for IgG inhibition of grass allergens in the combined and grass-only formulations, respectively, as measured by ELISA competition assays using sera from rabbits immunized with *P. pratense* allergoids. Upper red line, and lower blue line represent the specification limits established during validation (according to regulatory guidelines).

**
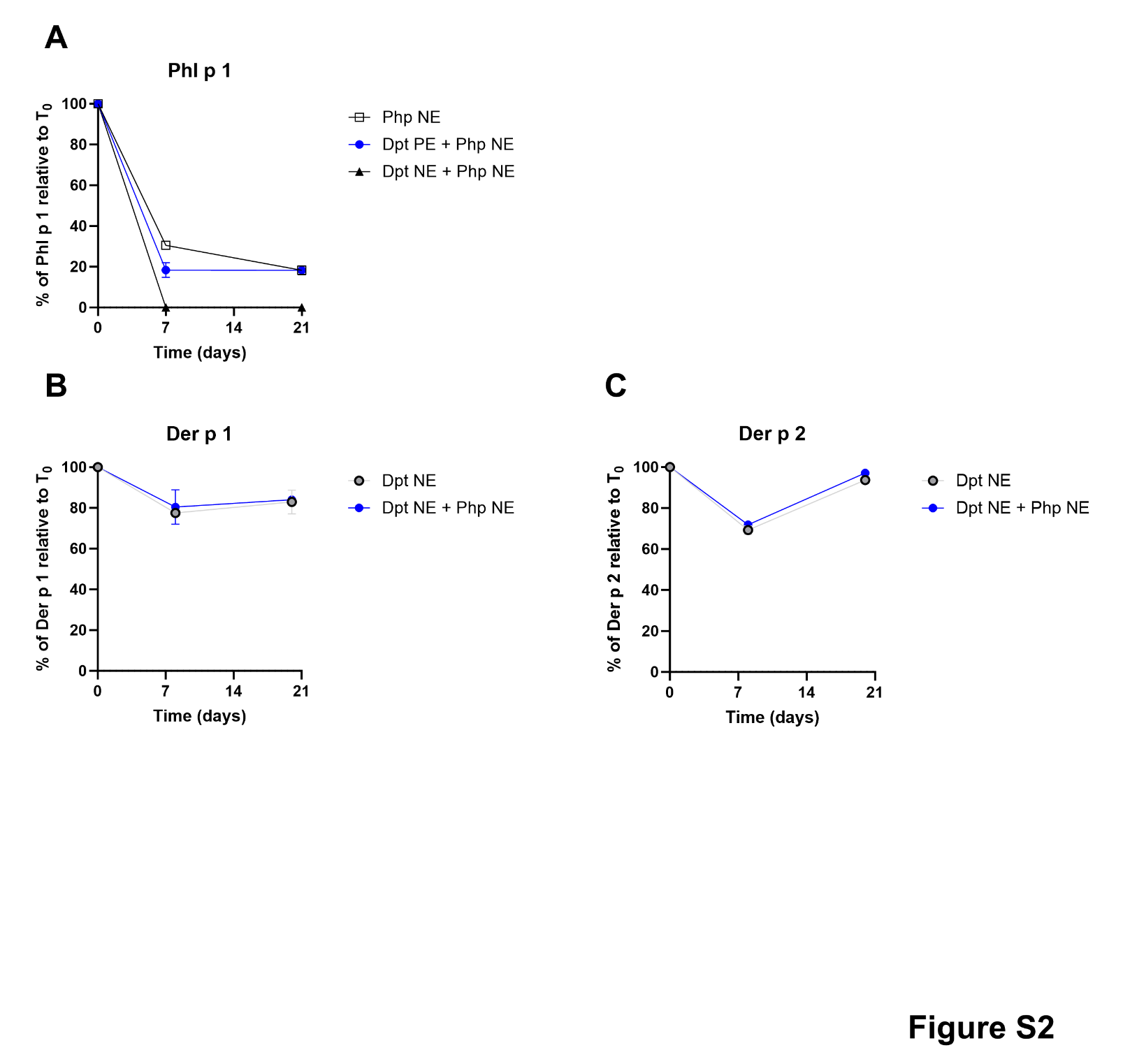
**

**Supplementary Figure 2. Quantification of Phl p 1 and major *D. pteronyssinus* allergens in mixtures or extracts alone**. Phl p 1 (A), Der p 1 (B) and Der p 2 (C) levels were quantified and expressed as a percentage relative to the initial amount at time 0 (T₀) in Php NE alone, (open squares), Dpt NE alone (grey circles), or mixtures, Php NE with Dpt PE (blue circles), and PhP NE with Dpt NE (black triangles). Error bars represent SEM from the mean. One representative experiment from two independent experiments.
